# Ultrasound Reference Chart Based on IVF Dates to Estimate Gestational Age at 6–9 weeks' Gestation

**DOI:** 10.5402/2012/938583

**Published:** 2012-07-22

**Authors:** Pavitra Delpachitra, Kirsten Palmer, Joseph Onwude, Simon Meagher, Luk Rombauts, Karen Waalwyk, Michael Bethune, Stephen Tong

**Affiliations:** ^1^Department of Obstetrics and Gynaecology, Monash University, Clayton, VIC 3168, Australia; ^2^Mercy Hospital for Women, University of Melbourne, Heidelberg, VIC 3084, Australia; ^3^Springfield Hospital, Lawn Lane, Essex, Chelmsford CM1 7GU, UK; ^4^Monash Ultrasound for Women, Monash Surgical Private Hospital, Clayton, VIC 3168, Australia; ^5^Monash IVF, Monash Surgical Private Hospital, Clayton, VIC 3168, Australia

## Abstract

Accurate determination of gestational age underpins good obstetric care. We assessed the performance of six existing ultrasound reference charts to determine gestational age in 1268 singleton IVF pregnancies, where “true” gestational age could be precisely calculated from date of fertilisation. All charts generated dates significantly different to IVF dates (*P* < 0.0001 all comparisons). Thus we generated a new reference chart, The Monash Chart, based on a line of best fit describing crown-rump length across 6 + 1 to 9 + 0 weeks of gestation (true gestational age) in the IVF singleton cohort. The Monash Chart, but none of the existing charts, accurately determined gestational age among an independent IVF twin cohort (185 twin pairs). When applied to 3052 naturally-conceived singletons scans, The Monash Chart generated estimated due dates that were different to all existing charts (*P* ≤ 0.004 all comparisons). We conclude that commonly used ultrasound reference charts have inaccuracies. We have generated a CRL reference chart based on true gestational age in an IVF cohort that can accurately determine gestational age at 6–9 weeks of gestation.

## 1. Introduction

Accurate dating of gestational age is central to good obstetric care. It allows the clinicians to better time gestation-specific antenatal screening tests, reduces erroneous labelling of pregnancies as very preterm, preterm, and small-for-gestational-age, and decreases the risk of inappropriate induction of labour [1–5].

In the first trimester, there is a very little biologic variation in fetal size compared with later trimesters. It is therefore a good time in pregnancy to determine gestational age by ultrasound where the crown-rump length (CRL) is measured and compared to published reference charts. In contrast, dates calculated from the first day of the last menstrual period (menstrual age) may have inaccuracies arising from imprecise recollection of dates, variation in the timing of ovulation, or time to conception.

A number of CRL reference charts have been proposed and different versions are in common use (Table 1). Most of these charts are based on menstrual age to estimate gestational age at the day of the ultrasound examination, based on modest sample sizes, generated many years ago using ultrasound machines of poorer resolution, or used transabdominal measurements (which gives poorer pictures relative to a transvaginal approach) [6–8]. As such, there is considerable variability between current reference charts, and estimated gestational age can vary significantly depending on which chart is used.

It should be possible to generate very accurate reference charts by using a large *in vitro* fertilisation (IVF) cohort where CRL measurements could be correlated with gestational age precisely calculated from date of fertilisation. Accuracy could be further enhanced by using measurements obtained from high-resolution transvaginal scans by sonologists specialising in Women's Health.

We examined CRL lengths at 6–9 weeks of gestation measured in a large IVF cohort where gestational age could be calculated from date of fertilisation. We assessed the ability of six commonly used reference charts to accurately date these pregnancies. Given all these charts showed inaccuracies in their ability to date these IVF pregnancies, we developed a new reference chart based on IVF dates in a singleton cohort.

## 2. Methods

### 2.1. Participants and Study Design

We retrospectively obtained clinical details on 1268 singleton IVF pregnancies conceived using a fresh embryo transfer cycle, had a transvaginal first trimester ultrasound done between 6 (+1d) to 9 (+0d) weeks of gestation where CRL were measured, and progressed to viability (>24 weeks of gestation). These were identified from a total cohort of 4971 first trimester ultrasound reports of IVF and naturally conceived pregnancy scans. Pregnancies that resulted from frozen embryos transfer, complicated by fetal structural anomalies, delivered before 24 weeks, or had missing data were all excluded. Of our cohort of 1268 pregnancies, 84 were scanned twice, and 2 pregnancies were scanned three times, giving a total population size of 1182. In these pregnancies with multiple scans, all data were included in the analysis as discrete values.

We restricted our analysis to those who had fresh embryo transfer since we were concerned with the need to add the time from egg pickup to freezing, together with the time from subsequent thawing to transfer might introduce inaccuracies. We determined gestational age on the day of the ultrasound (IVF dates) by nominating the day of egg pickup and fertilisation as day 14 of gestation.

We first compared IVF dates with estimated dates determined using six existing reference charts: Australian Society for Ultrasound Medicine (ASUM) [10], Hadlock et al. [11], Daya [6], Verburg et al. [9], Old ASUM and Robinson [12, 13]. Note that some charts did not have corresponding gestational ages for all measurements which accounts for the variability in sample size seen in the comparison of charts. We then generated reference charts using the CRL measurements and IVF dates by smoothing out the data (see statistical analysis below), which we named The Monash Chart.

To validate our chart, we obtained CRL measurements from an IVF twin cohort (fresh embryo transfer) at 6 (+1d) to 9 (+0d) weeks of gestation where gestational age was calculated (fertilisation age + 14 days). Taking each twin as a discrete measurement, we determined the accuracy of all six existing reference charts and The Monash Chart in estimating gestational age.

We then applied all six existing reference charts and our chart to estimate gestational age of CRL measurements obtained from 3052 consecutive first-trimester singleton ultrasound scans pregnancies at 6–9 weeks of gestation.

Ethics approval was obtained before we commenced the study (Project 05063, Monash Surgical Private Human Research Ethics Committee, Clayton, VIC, Australia). For this retrospective database study where we used de-identified data in aggregate, the ethics committee specifically approved our request not to obtain individual patient consent.

### 2.2. Ultrasound Examinations

All examinations were performed at three ultrasound centres that exclusively perform women's health ultrasounds. All ultrasounds were transvaginal, done on Advanced Technology Laboratories^T^ HDI 5000 ultrasound machines by experienced sonographers. After confirmation of a live intrauterine pregnancy, the CRL was measured in the midsagittal plane by the placement of ultrasound callipers at the outer edges of the head and rump of the fetus, excluding the limbs and yolk sac. Two measurements were taken, with the average taken as the final measurement.

### 2.3. Statistics

For comparison of data, an unpaired Student's *t*-test was used to compare two groups with continuous variables that were normally distributed and nonparametric data was compared using Mann-Whitney *U* test. Nonparametric data was expressed as median and interquartile range, while parametric data was expressed as mean (±standard deviation).

**Figure 1:**
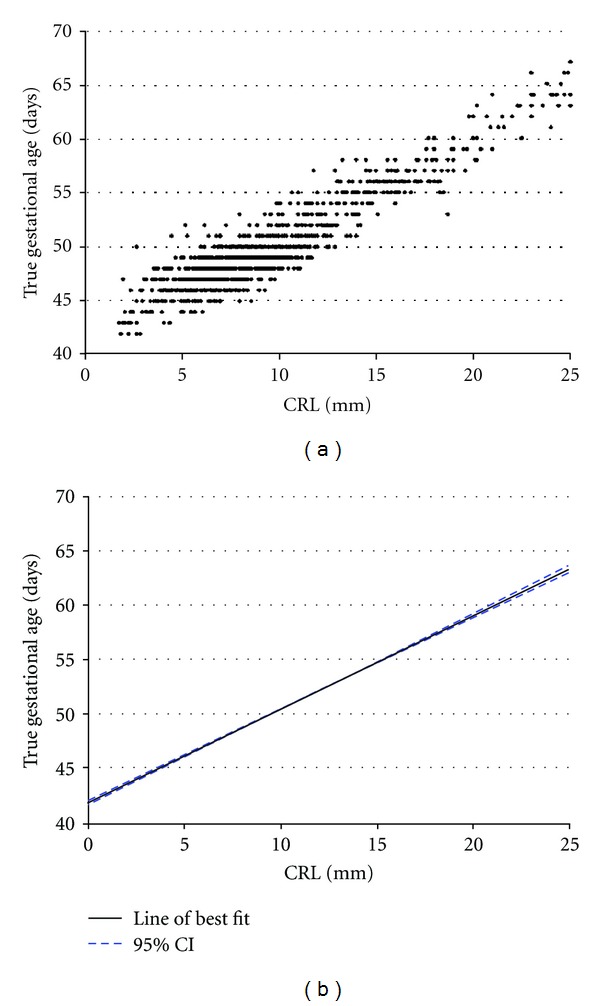
(a) scatterplot and (b) line of best fit (black line shows line of best fit, and blue line shows ±95% confidence interval) to describe CRL length graphed against “true” gestational age (calculated from date of conception in the IVF singleton cohort). *n* = 1268.

To determine the relationship between true gestational age and CRL, we constructed a scattergram, plotting CRL lengths against true gestational age in our singleton IVF cohort (Figure 1(a)). Investigation using fractional polynomial regression analysis [14] revealed that a straight line best described the mean. The standard deviation (SD) varied very little at every week of gestational age, which was unsurprising given our significant cohort size. The SD was therefore termed as a constant (the residual standard deviation). The “goodness of fit” was determined by a plot of the standard deviation score or standardised residual which was normally distributed. To develop The Monash Chart, a linear prediction plot was fitted to the scattergram (Figure 1(a)) and appeared to be the best model to fit the data with a very narrow 95% confidence interval (Figure 1(b)). The final CRL reference chart was derived from the equation describing the line of best fit.

When we compared the six existing CRL reference charts to either IVF dates or to gestational ages derived for The Monash chart, we calculated the mean differences of the gestational ages from the six charts from either the IVF true gestational age or The Monash Chart gestational age (depending on the analysis being undertaken), and compared them with paired *t*-tests.

## 3. Results

### 3.1. Accuracy of Existing Reference Charts

We identified 1268 first trimester ultrasound scans done at 6 (+1d) to 9 (+0d) weeks of gestation where gestational age at the day of the ultrasound could be precisely determined using the IVF dates. We calculated IVF dates by noting the number of days from fertilisation until the date of the ultrasound assessment. Since day of egg pickup is day 14 of gestation by convention, an extra 14 days were added to this number in order to calculate the IVF dates.

The clinical characteristics of this IVF cohort are shown in Table 2. We noted the raw CRL measurement in millimetres and compared gestational age estimated by six existing CRL reference charts with actual IVF dates. We found the mean gestational age estimated by all these charts varied significantly to IVF dates (*P* < 0.0001 for all charts), with mean dates varying from −1.2 to 2.1 days (Table 3).

### 3.2. Construction of New Chart Based on True Gestational Age

We plotted CRLs against gestational age determined by IVF dating on a scattergram (Figure 1(a)). After using fractional polynomial regression analysis [14], we found a straight line best described the mean. We determined a line of best fit with a 95% confidence interval noted to be very narrow across the entire gestational age range (Figure 1). Using the equation that describes this line (Gestational age = 0.82 [CRL in mm] + 42.1), we derived a new CRL reference chart based on IVF dates (Table 4) that we termed The Monash Chart.

To ensure the generation of a line of best fit to create The Monash Chart did not significantly distort prediction of gestational age, we compared estimated gestational age calculated from our chart with actual IVF dates. No difference was seen (*P* = 0.2264). We then compared gestational age derived from The Monash Chart with that predicted by the six existing reference charts and found significant differences for all charts (mean difference in estimated gestational age varied from −1.3 days to 2.1 days; *P* < 0.0001 for all charts). 

### 3.3. Validation of the Monash Chart in an IVF Twin Cohort

We next sought to validate The Monash Chart. To do this, we chose an independent IVF twin pregnancy (*n* = 185 twin pairs, or 370 independent observations) conceived after fresh embryo transfer where exact gestational age (fertilisation age + 14 days) was known, and the result was delivery of two babies >24 weeks of gestation. We used a twin cohort to validate our chart for two reasons. First, it would validate the use of our chart for twins, a situation where calculation of estimated delivery dates is especially important given obstetric risks. Secondly, the use of IVF pregnancies again allows accurate determination of gestational age by calculating IVF dates. While there may be differences in growth between twins and singletons in late pregnancy, there is no evidence that differences in CRL between singletons and twins exist. Biological differences in CRL as large as millimetres at this early gestation would be very unlikely.

The mean (SD) maternal age of the IVF twin cohort was 33 (3.9) years, mean (SD) gestation at birth was 36 (2.5) weeks' gestation. Mean birthweight for twin 1 was 2492 (548) gms and twin 2 was 2467 (576) gms. Median (range) treatment cycle number was 3 (1–15) and the median (range) number of embryos transferred was 2 (1–3).

In this cohort of IVF twins, predicted gestational age from the six existing reference charts was significantly different from IVF dates, with mean differences ranging from −1.1 days to 2.3 days (*P* ≤ 0.0005). Only The Monash Chart was not statistically significantly different to IVF dates in the twin cohort (*P* = 0.6835).

### 3.4. Application of Chart to an Unselected Singleton Population

We next applied our chart to CRL measurements obtained from 3052 consecutive first-trimester viability ultrasounds. The purpose was to see whether The Monash Chart would pragmatically alter expected dates of delivery compared to existing charts. We found dates derived from our chart were significantly different to the existing reference charts (mean difference in estimated gestation ranged from −1.8 days to 1.8 days; *P* ≤ 0.0047 for all charts, see Table 5).

## 4. Discussion

Many women who have a positive pregnancy test request an ultrasound to confirm viability. Therefore, early pregnancy “viability” ultrasounds performed at 6–9 weeks' gestation are done very often, where gestational age is derived from the CRL measurements.

We have developed a potentially highly accurate CRL reference chart to date pregnancies at the viability ultrasound. The Monash Chart is based on IVF dates. While others have proposed CRL charts based on IVF dates before, they have been based on small numbers (36–160 participants) [6, 15–18]. In contrast, ours was generated from a more sizable population (*n* = 1268). Given a possible association between shorter than expected CRL and miscarriage [19], we only included pregnancies that progressed beyond 24 weeks' gestation. We validated the performance of our chart using an independent twin cohort, and showed in a further cohort of 3052 consecutive ultrasounds it would materially alter dates if it used instead of any of six preexisting charts. Hence we believe our chart may possibly be the most accurate of any published chart to date pregnancies between 6–9 weeks of gestation.

In addition, we found inaccuracies in the ability of commonly used charts to estimate gestational age among IVF singleton and twin cohorts where exact dates are known. While some showed only very slight differences in the estimation of dates compared to IVF dates (e.g., 0.57 days mean difference for Daya chart) and others showed larger differences (2.1 days for ASUM chart), all were highly statistically different (Table 3). Of further concern is the fact that there appears to be significant disagreement between existing charts where some given CRL lengths, predicted gestational age can vary by many days depending on which chart is referenced.

In order to determine precisely gestational age on the day of the ultrasound scan, we have necessarily derived our reference chart from an IVF population. While there may be some differences in final birthweight among those conceived by IVF compared to spontaneous conceptions [20], there is no evidence to suggest differential CRLs exist between these two groups. Given variations of even a millimetre or two would represent significant proportional differences in length at these early gestations, we consider it unlikely The Monash Chart would not be valid for spontaneously conceived pregnancies.

In this study, we were unable to generate a reference chart that encompassed CRL reference ranges across the whole first trimester. The reason is that CRL measurements were rarely performed across 9–11 weeks of gestation among our IVF cohort. The likely reason is that being IVF pregnancies, clinicians had exact dates with which to time the late first trimester ultrasound to 12+ weeks of gestation, when nuchal translucency is best assessed. We attempted modelling a chart incorporating these late first trimester CRL lengths, but we could not be confident that the integrity and high accuracy of the 6–9 week chart we report was maintained. Also, we did not include gestations under 6 weeks given CRL at (6+1) is already just 1 mm, and it is not possible for ultrasound to accurately measure differential CRL lengths present at earlier gestations. Nevertheless, we believe our chart is still clinically useful since many spontaneous pregnancies will have the first ultrasound between 6–9 weeks of gestation.

Strengths of our study includes the fact we only used measurements obtained from high-resolution transvaginal ultrasounds of CRLs at centres that exclusively perform obstetrics and gynecological ultrasounds. Also, we utilized a large cohort, reflected by the fact that the 95% confidence intervals are very narrow (Figure 1).

Accurate dating is important since obstetric management throughout pregnancy is strongly based on gestational age. For instance, the first trimester nuchal translucency measurements are most accurate if performed during the 12th week of gestation [21]. Many units offer an induction of labour at exactly ten to fourteen days after the expected date of delivery, and no later given concerns that stillbirth rates may increase more steeply beyond two weeks after the expected date of delivery [22]. A Cochrane systematic review concluded that accurate dating indeed reduces the rates of induction of labour for postdates [23].

At the thresholds of viability, a matter of days can sometimes impact on clinical decisions. Many would offer conservative management if a delivered baby is judged to be around 23 weeks +3 days of gestation, but may consider actively resuscitating a baby estimated to have reached 24 weeks +2 days of gestation. Therefore, it is important to be as accurate as possible in determining gestational age.

In conclusion, we have generated The Monash Chart that we believe may be the most accurate CRL chart reference chart yet proposed to date pregnancies at 6–9 weeks' gestation. Furthermore, our chart appears valid for both singleton and twin pregnancies.

## Figures and Tables

**Table 1:** Approaches used by six previous studies to generate CRL reference charts.

	Robinson	Robinson/Fleming	Hadlock	Daya	Westerway (ASUM)	Verburg
Year	1973	1976	1992	1993	2000	2008
Probe used	TA	TA	TA	TA, TV	TA, TV	TA, TV
Method to calculate gestation at ultrasound	LMP	LMP	LMP	IVF dates	LMP	LMP
*n* ^ ∗^	214 (80)	334	452 (416)	94	478	3760
Gestational ages that reference ranges were reported (weeks)	6–14	6–14	5.7–18	6.1–13.3	5.2–14.4	6–15

Scan: type of ultrasound scanner used, technique: technique used for scanning, TA: transabdominal, TV: transvaginal, RT: real time, LMP: last menstrual period, IVF: *in vitro* fertilisation. ^∗^ *n* represents the number of observations, number of patients in brackets if this is different to number of observations.

**Table 2:** Baseline characteristics of study participants the IVF singleton cohort.

Baseline characteristics—Singleton IVF cohort *n* = 1268	
Age (years)—mean (SD)	34.3 (4.28)
Gestation at birth (weeks)—mean (SD)	38.6 (2.15)
Birthweight (grams)—mean (SD)	3266 (603.2)
Treatment cycle—median (range)	2 (1–38)
Embryos transferred—median (range)	2 (1–3)

SD: standard deviation.

**Table 3:** Estimated gestational age of 1268 fetuses derived from six existing CRL reference charts compared to IVF dates.

	*n*	Range of differences to true gestational age in days	Mean difference (95% CI) in days	Comparison with true gestational age
ASUM	1233^∗^	−10.5 to 8	2.1 (2.0 to 2.2)	*P* < 0.0001
Hadlock	1268	−9 to 8	1.2 (1.1 to 1.3)	*P* < 0.0001
Daya	1268	−8 to 9	−0.57 (−0.67 to −0.48)	*P* < 0.0001
Verburg	271^∗^	−8 to 10	−1.2 (−1.5 to −0.81)	*P* < 0.0001
Old ASUM	1263^∗^	−9 to 8	0.43 (0.33 to 0.53)	*P* < 0.0001
Robinson	636^∗^	−7 to 10	0.39 (0.21 to 0.57)	*P* < 0.0001

A negative number denotes the number of days that estimated gestational age lagged behind true gestational age. ^∗^ *n* values are less than 1268 because exact dates were not given for a number of CRL lengths in these charts. Verburg et al. [9], for instance, only provide exact dates for CRL lengths at 5, 10, and 15 mm.

**Table 4:** The Monash Chart. CRL reference table based on true gestational age in an IVF cohort.

CRL (mm)	GA (wks, days)	GA (days)
1.0 mm	6W1D	42.9
1.5 mm	6W1D	43.3
2.0 mm	6W2D	43.7
2.5 mm	6W2D	44.1
3.0 mm	6W3D	44.5
3.5 mm	6W3D	45.0
4.0 mm	6W3D	45.4
4.5 mm	6W4D	45.8
5.0 mm	6W4D	46.2
5.5 mm	6W5D	46.6
6.0 mm	6W5D	47.0
6.5 mm	6W5D	47.4
7.0 mm	6W6D	47.8
7.5 mm	6W6D	48.2
8.0 mm	7W0D	48.6
8.5 mm	7W0D	49.1
9.0 mm	7W0D	49.5
9.5 mm	7W1D	49.9
10.0 mm	7W1D	50.3
10.5 mm	7W2D	50.7
11.0 mm	7W2D	51.1
11.5 mm	7W3D	51.5
12.0 mm	7W3D	51.9
12.5 mm	7W3D	52.3
13.0 mm	7W4D	52.7
13.5 mm	7W4D	53.2
14.0 mm	7W5D	53.6
14.5 mm	7W5D	54.0
15.0 mm	7W5D	54.4
15.5 mm	7W6D	54.8
16.0 mm	7W6D	55.2
16.5 mm	8W0D	55.6
17.0 mm	8W0D	56.0
17.5 mm	8W0D	56.4
18.0 mm	8W1D	56.8
18.5 mm	8W1D	57.3
19.0 mm	8W2D	57.7
19.5 mm	8W2D	58.1
20.0 mm	8W2D	58.5
20.5 mm	8W3D	58.9
21.0 mm	8W3D	59.3
21.5 mm	8W4D	59.7
22.0 mm	8W4D	60.1
22.5 mm	8W5D	60.5
23.0 mm	8W5D	60.9
23.5 mm	8W5D	61.4
24.0 mm	8W6D	61.8
24.5 mm	8W6D	62.2
25.0 mm	9W0D	62.6
25.5 mm	9W0D	63.0
26.0 mm	9W0D	63.4

GA: gestational age, W: weeks, and D: days.

**Table 5:** Mean differences in gestational ages derived from The Monash Chart compared to those predicted by the six existing reference charts.

	*n*	Mean differences in days (95% CI)	Comparison with gestation determined using The Monash Chart
ASUM	2938^∗^	1.8 (1.7 to 1.9)	*P* < 0.0001
Hadlock	3052	0.87 (0.81 to 0.93)	*P* < 0.0001
Daya	3052	−0.80 (−0.84 to −0.77)	*P* < 0.0001
Verburg	641^∗^	−1.80 (−2.0 to −1.6)	*P* < 0.0001
Old ASUM	2997^∗^	0.23 (0.20 to 0.27)	*P* < 0.0001
Robinson	1476^∗^	−0.16 (−0.27 to −0.05)	*P* = 0.0047

^∗^ *n* values are less than 3052 because exact dates were not given for a number of CRL lengths in these charts. Verburg et al. [9], for instance, only provide exact dates for CRL lengths at 5, 10, and 15 mm.

## References

[1] McGalliard C, Gaudoin M (2004). Routine ultrasound for pregnancy termination requests increases women’s choice and reduces inappropriate treatments. *An International Journal of Obstetrics and Gynaecology*.

[2] Wald NJ, Cuckle HS, Densem JW, Kennard A, Smith D (1992). Maternal serum screening for Down’s syndrome: the effect of routine ultrasound scan determination of gestational age and adjustment for maternal weight. *British Journal of Obstetrics and Gynaecology*.

[3] Blondel B, Morin I, Platt RW, Kramer MS, Usher R, Bréart G (2002). Algorithms for combining menstrual and ultrasound estimates of gestational age: consequences for rates of preterm and postterm birth. *An International Journal of Obstetrics and Gynaecology*.

[4] Reuss ML, Hatch MC, Susser M (1995). Early ultrasound dating of pregnancy: selection and measurement biases. *Journal of Clinical Epidemiology*.

[5] Taipale P, Hiilesmaa V (2001). Predicting delivery date by ultrasound and last menstrual period in early gestation. *Obstetrics and Gynecology*.

[6] Daya S (1993). Accuracy of gestational age estimation by means of fetal crown-rump length measurement. *American Journal of Obstetrics and Gynecology*.

[7] Kalish RB, Thaler HT, Chasen ST (2004). First- and second-trimester ultrasound assessment of gestational age. *American Journal of Obstetrics and Gynecology*.

[8] McLennan AC, Schluter PJ (2008). Construction of modern Australian first trimester ultrasound dating and growth charts. *Journal of Medical Imaging and Radiation Oncology*.

[9] Verburg BO, Steegers EAP, De Ridder M (2008). New charts for ultrasound dating of pregnancy and assessment of fetal growth: longitudinal data from a population-based cohort study. *Ultrasound in Obstetrics and Gynecology*.

[10] Westerway SC, Davison A, Cowell S (2000). Ultrasonic fetal measurements: new Australian standards for the new millennium. *Australian and New Zealand Journal of Obstetrics and Gynaecology*.

[11] Hadlock FP, Shah YP, Kanon DJ, Lindsey JV (1992). Fetal crown-rump length: reevaluation of relation to menstrual age (5–18 weeks) with high-resolution real-time US. *Radiology*.

[12] Robinson HP (1973). Sonar measurement of fetal crown rump length as means of assessing maturity in first trimester of pregnancy. *British Medical Journal*.

[13] Robinson HP, Fleming JEE (1975). A critical evaluation of sonar ’crown rump length’ measurements. *British Journal of Obstetrics and Gynaecology*.

[14] Royston P, Wright EM (1998). How to construct ’normal ranges’ for fetal variables. *Ultrasound in Obstetrics and Gynecology*.

[15] Silva PD, Mahairas G, Schaper AM, Schauberger CW (1990). Early crown-rump length. A good predictor of gestational age. *Journal of Reproductive Medicine for the Obstetrician and Gynecologist*.

[16] MacGregor SN, Tamura RK, Sabbagha RE (1987). Underestimation of gestational age by conventional crown-rump length dating curves. *Obstetrics and Gynecology*.

[17] Wisser J, Dirschedl P, Krone S (1994). Estimation of gestational age by transvaginal sonographic measurement of greatest embryonic length in dated human embryos. *Ultrasound in Obstetrics & Gynecology*.

[18] Sladkevicius P, Saltvedt S, Almström H, Kublickas M, Grunewald C, Valentin L (2005). Ultrasound dating at 12-14 weeks of gestation. A prospective cross-validation of established dating formulae in in-vitro fertilized pregnancies. *Ultrasound in Obstetrics and Gynecology*.

[19] Mukri F, Bourne T, Bottomley C, Schoeb C, Kirk E, Papageorghiou AT (2008). Evidence of early first-trimester growth restriction in pregnancies that subsequently end in miscarriage. *An International Journal of Obstetrics and Gynaecology*.

[20] Schieve LA, Meikle SF, Ferre C, Peterson HB, Jeng G, Wilcox LS (2002). Low and very low birth weight in infants conceived with use of assisted reproductive technology. *New England Journal of Medicine*.

[21] Ndumbe FM, Navti O, Chilaka VN, Konje JC (2008). Prenatal diagnosis in the first trimester of pregnancy. *Obstetrical and Gynecological Survey*.

[22] Divon MY, Feldman-Leidner N (2008). Postdates and antenatal testing. *Seminars in Perinatology*.

[23] Whitworth M, Bricker L, Neilson JP, Dowswell T (2010). Ultrasound for fetal assessment in early pregnancy.. *Cochrane Database of Systematic Reviews*.

